# Numerical Study on the Solidification Microstructure Evolution in Industrial Twin-Roll Casting of Low-Carbon Steel

**DOI:** 10.3390/ma18194484

**Published:** 2025-09-26

**Authors:** Yulong Shi, Kongfang Feng, Liang Liu, Gaorui He, Bo Wang

**Affiliations:** 1 School of Materials Science and Engineering, Shanghai University, Shanghai 200444, China; 2 State Key Laboratory of Advanced Special Steel, Shanghai University, Shanghai 200444, China; 3 Institute of Research of Iron & Steel, Shasteel, Zhangjiagang 215625, China

**Keywords:** twin-roll strip casting, microstructure, delivery system, side hole, average grain size

## Abstract

Twin-roll strip casting (TRSC) is a key development in near-net-shape casting technology, offering the potential for high-efficiency and low-cost production. During the TRSC process, the solidification characteristics of the strip are largely governed by the configuration of the melt delivery system as well as by various process parameters. In this study, a three-dimensional model of low-carbon steel strip casting was developed using ProCAST software to investigate microstructure evolution under industrial-scale conditions. Simulation results revealed that the solidified strip exhibits a typical three-layer structure: a surface equiaxed grain zone in contact with the cooling rolls, a subsurface columnar grain zone, and a central equiaxed grain zone. Introducing side holes into the delivery system promoted the formation of a distinct columnar grain region near the side dams, resulting in a reduction in the average grain size in this region from 43.7 μm to 38.2 μm compared to the delivery system without side holes. Increasing the heat transfer coefficient at the interface between the molten pool and the cooling rolls significantly enlarged the columnar grain zone. This change had little effect on the average grain size and grain density, with the average grain size remaining close to 37 μm and the grain density variation being less than 0.7%. In contrast, when the casting speed was raised from 50 m min^−1^ to 70 m min^−1^, a reduction in the area of the columnar grain zone was observed, while the average grain size decreased slightly (by less than 0.5 μm), and the grain density increased accordingly. This study provides valuable insights for optimizing process parameters and designing more effective melt delivery systems in industrial twin-roll strip casting.

## 1. Introduction

Twin-roll strip casting represents a near-net-shape production technique in which sub-rapid solidification and hot deformation occur simultaneously. In contrast with traditional continuous casting methods, it offers advantages such as a shorter process route, lower energy consumption, and reduced production costs. It is regarded as one of the most promising and revolutionary metallurgical technologies of the 21st century. In the twin-roll strip casting process, molten metal is continuously fed through a delivery system into a molten pool confined by two counter-rotating water-cooled rolls and lateral side dams [1,2]. The molten metal solidifies on the surface of the cooling rolls to form solid shells, which are subsequently compressed at the roll nip to produce thin strips, typically on the millimeter scale in thickness [3,4]. This unique combination of rapid solidification and high-temperature deformation significantly reduces grain size and suppresses solute segregation. However, due to the limited thickness of the as-cast strip, post-processing is constrained, and any defect introduced during solidification, such as uneven grain morphology, macrosegregation, or cracks, can critically impair product quality and yield. Therefore, optimizing the solidification behavior becomes essential to ensure process reliability and product integrity. This stringent process requirement aligns with the global pursuit of green and sustainable industrial development. The twin-roll strip casting process, with its short process flow, fast solidification rate, and low energy consumption, has become a focal point of innovation in the metallurgical industry [5,6,7,8]. Meanwhile, carbon content, as a key factor influencing the microstructure and properties of steel materials, as well as an important variable affecting energy consumption and carbon emissions during production, plays a significant role. Under the drive of the “dual-carbon” goals, the coordinated optimization of carbon content control and continuous casting process innovation can achieve the efficiency and greening of the entire process chain, thereby promoting the development of the metallurgical industry toward a low-carbon and sustainable direction [9].

The solidification microstructure in twin-roll casting is strongly affected by thermal and fluid flow conditions, which are primarily influenced by the configuration of the delivery system as well as critical process parameters, including casting speed, superheat, and roll cooling intensity. Extensive research has been carried out to understand these effects using both experimental and numerical approaches. Experimental studies remain the most direct approach for investigating the solidification microstructure in twin-roll strip casting. Although laboratory-scale casters are limited in size and cannot fully replicate industrial-scale strip structures, they effectively reveal the influence of processing parameters on solidification behavior. Fang et al. [10] investigated the solidification microstructure of silicon steel via experiments and found that increasing the casting temperature enlarged grain size and increased the proportion of columnar grains. Across the strip thickness, equiaxed grains appear at the surface, followed by a sub-surface columnar grain zone and central equiaxed grains. Along the strip width, the distribution of columnar and equiaxed grains also varies. Process parameters significantly influence the microstructure evolution during twin-roll casting. Han et al. [11] compared the effects of copper and steel rolls on the solidification of non-oriented silicon steel. Strips cast with copper rolls showed a three-layer microstructure similar to Fang’s results, while the lower thermal conductivity of steel rolls led to slower cooling and the formation of coarser, fully equiaxed grains.

In addition to experimental studies, computational modeling has emerged as an effective method for exploring solidification phenomena in twin-roll strip casting. It enables detailed analysis of temperature distribution, solidification front evolution, and microstructure formation under various process conditions, which are often difficult to capture experimentally. Yang et al. [12] developed a comprehensive numerical model for twin-roll strip casting of stainless steel by modifying the KGT model, incorporating heterogeneous nucleation and columnar-to-equiaxed transition mechanisms. With this model, the influences of casting temperature, withdrawal speed, and melt pool height on the columnar grain fraction, dendrite arm spacing, and grain orientation were systematically evaluated. Lv Zheng [13], Zhang Dongxiao [14], Liu Haitao [15], and Hu Yan [16] used two-dimensional thermal-flow models to investigate the effect of process parameters on the solidification microstructure. The results showed that higher casting speed reduces columnar grain zones and grain size, while greater superheat or stronger cooling increases the fraction of equiaxed grains. Building on these efforts, Wang et al. [17] developed a more detailed three-dimensional CAFE model using the CALCOSOFT3D package. Their simulation revealed that pouring temperature had a stronger influence on grain density than interfacial heat transfer conditions, underscoring the key role of thermal parameters in microstructure evolution.

Although a large body of work has examined the solidification features and transport behavior of twin-roll strip casting through experiments and modeling, several challenges remain. However, most existing studies rely either on small-scale experiments that do not reflect industrial conditions or on two-dimensional models that fail to capture spatial variations across the strip. To overcome these limitations, a comprehensive three-dimensional model incorporating the melt delivery system and molten pool was developed based on an industrial twin-roll strip caster. The effects of delivery system configuration and process parameters on solidification behavior and microstructure evolution are systematically explored using the ProCAST software [18,19]. These works provide a more realistic simulation approach under industrial conditions and offer valuable insights for process optimization and the design of efficient melt delivery systems.

## 2. Physical and Mathematical Models of Twin-Roll Strip Casting

### 2.1. Geometric Configuration and Physical Model

**Figure 1:**
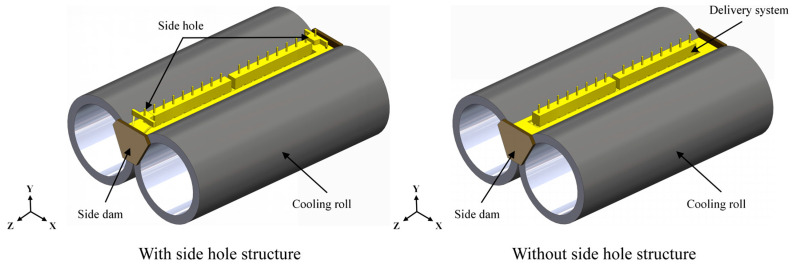
Schematic of the melt delivery system and molten pool in twin-roll strip casting.

Figure 1 provides a detailed physical representation of the twin-roll strip casting process consisting of the melt delivery system and the molten pool. As illustrated, two configurations of the delivery system are considered: with side holes and without side holes. Molten steel is directed through the delivery system into a molten pool, which is confined by a pair of counter-rotating cooling rolls and side dams. The gap between the two cooling rolls is approximately 1.5 mm, with each roll measuring 500 mm in diameter and 1345 mm in length. The molten steel level in the pool is maintained at 175 mm. In the configuration with side holes, liquid steel is fed into the molten pool from above at an angle, near the side dams, inducing a more vigorous flow pattern in these regions and enhancing fluid motion near the side dams.

**Figure 2:**
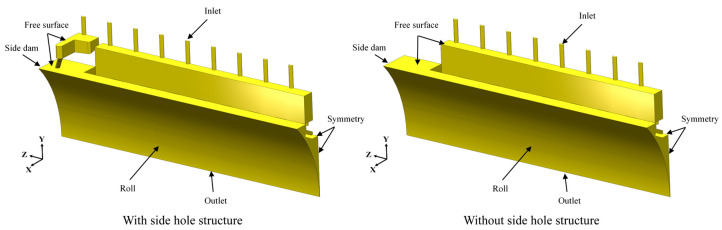
Quarter-section numerical simulation model of the twin-roll strip casting process.

To improve computational efficiency, symmetry in both the strip width (Z-axis) and thickness (X-axis) directions is utilized, allowing only one-quarter of the domain to be modeled numerically, as shown in Figure 2.

### 2.2. Macroscopic Thermal–Fluid Model 

Throughout the twin-roll strip casting operation, molten steel enters the narrow gap between two counter-rotating cooling rolls, forming a confined molten pool where solidification takes place under intense thermal and mechanical conditions. Within the molten pool, steel flow is characterized by turbulence and temporal fluctuations, arising from rapid casting and steep temperature gradients [20,21,22].

The macroscopic behavior of fluid flow, heat transfer, and solidification in the molten pool is governed by a set of fundamental conservation equations, including the continuity equation, momentum equations (Navier–Stokes), turbulence model equations, and the energy conservation equation. The following equations form the basis of the macroscopic thermal–fluid model:

#### 2.2.1. Mass Conservation Equation

In fluid dynamics, the law of mass conservation must always be satisfied. For molten steel considered as an incompressible medium, this conservation relationship is described by the continuity equation as follows:

(1) $$\frac{\partial \rho }{\partial t}+\frac{\partial }{\partial x_{i}}(\rho u_{i})=0$$

where *ρ* is the density of molten steel (kg m^−3^), *t* is time (s), *x_i_* is the spatial coordinate in the *i*-direction (m), and *u_i_* is the velocity component in the *i*-direction (m s^−1^); where *ρ* stands for the molten steel density (kg m^−3^), *t* indicates the elapsed time (s), *x_i_* designates the spatial position in the *i*-th direction (m), and *u_i_* denotes the velocity component along that direction (m s^−1^).

#### 2.2.2. Momentum Conservation Equation

The momentum conservation equation describes the variation of fluid momentum under the influence of pressure, viscosity, and other forces. It is given by:

(2) $$\frac{\partial }{\partial t}(\rho u_{i})+\frac{\partial (\rho u_{i}u_{j})}{\partial x_{j}}=\rho g-\frac{\partial P}{\partial x_{i}}+\frac{\partial }{\partial x_{i}}[\mu_{\mathrm{eff}}(\frac{\partial u_{i}}{\partial x_{j}}+\frac{\partial u_{j}}{\partial x_{i}})]+\frac{u_{1}}{K_{p}}(u_{i}-u_{pi})$$

where *u_i_* and *u_j_* are the velocity components in the *i*- and *j*-directions (m s^−1^), *x_i_* and *x_j_* are the spatial coordinates in the *i*- and *j*-directions (m), *P* is the pressure (Pa), *µ_eff_* is the effective viscosity, *μ_l_* is the laminar viscosity, *K_p_* is the permeability (m^−2^), and *u_pi_* is the velocity component of the solidified shell in the *i*-direction (m s^−1^).

#### 2.2.3. Standard k–ε Turbulence Model

Turbulence within the molten pool is modeled using the standard k–ε formulation. The approach incorporates a pair of transport equations describing the evolution of turbulent kinetic energy (*k*) and its dissipation rate (*ε*), and these equations can be expressed as:

(3) $$\mu_{t}=\rho C_{\mu }\frac{k}{\varepsilon }$$

(4) $$\frac{\partial (\rho k)}{\partial t}+\frac{\partial (\rho u_{i}k)}{\partial x_{i}}=\frac{\partial }{\partial x_{i}}[(\mu +\frac{\mu_{t}}{\sigma_{k}})\frac{\partial k}{\partial x_{i}}]+\mu_{t}\frac{\partial u_{i}}{\partial x_{j}}(\frac{\partial u_{i}}{\partial x_{j}}+\frac{\partial u_{j}}{\partial x_{i}})-\rho \varepsilon +\frac{u_{1}}{K_{p}}k$$

(5) $$\frac{\partial }{\partial t}(\rho \varepsilon )+\frac{\partial (\rho \varepsilon u_{i})}{\partial x_{i}}=-\frac{\partial }{\partial x_{i}}(\mu +\frac{\mu_{t}}{\sigma_{\varepsilon }}\frac{\partial \varepsilon }{\partial x_{i}})+C_{1}\frac{\varepsilon }{k}\mu_{t}(\frac{\partial u_{i}}{\partial x_{j}}+\frac{\partial u_{j}}{\partial x_{i}})\frac{\partial u_{i}}{\partial x_{j}}-C_{2}\frac{\varepsilon^{2}}{k}+\frac{\mu_{1}}{K_{p}}\varepsilon$$

In the above equations, *k* and *ε* represent the turbulent kinetic energy (m^2^ s^−2^) and the turbulent dissipation rate (m^2^ s^−3^), respectively. *C*_1_, *C*_2_, *σ_k_*, *σ_ε_*, and *C_µ_* are empirical constants. The widely adopted values recommended by Launder and Spalding are 1.44, 1.92, 1.0, 1.3, and 0.09, respectively. To account for the influence of the mushy zone and solid phase on the flow behavior of molten steel in the pool, damping source terms are introduced into Equations (4) and (5).

#### 2.2.4. Energy Conservation Equation

The energy conservation equation describes the heat transfer process during fluid flow, including sensible heat and latent heat exchange during solidification. It can be expressed as:

(6) $$\rho \frac{\partial H}{\partial t}+\frac{\partial }{\partial x_{i}}(\rho u_{i}H)=\frac{\partial }{\partial x_{i}}(K_{eff}\frac{\partial T}{\partial x_{i}})$$

(7) $$H=H_{0}+\int_{T_{0}}^{T}C_{p}dT+(1-f_{s})L$$

(8) $$K_{eff}=K_{0}+K_{t}$$

(9) $$K_{t}=\frac{C_{p}\mu_{t}}{P_{r}}$$

where *H* is the enthalpy (J kg^−1^), *H*_0_ is the reference enthalpy at temperature *T*_0_ (J kg^−1^); *T_0_* is the instantaneous temperature of the molten steel (K); *C_p_* is the specific heat capacity (J kg^−1^ K^−1^); *L* is the latent heat of solidification (J kg^−1^); *f_s_* is the solid fraction of molten steel; *K_eff_* is the effective thermal conductivity (W m^−1^ K^−1^), composed of the molecular thermal conductivity *K*_0_ and turbulent conductivity; *μ_t_* is the turbulent viscosity (Pa s); and *P_r_* is the turbulent Prandtl number.

Solidification is modeled using the enthalpy method, which accounts for the latent heat release during phase change. The model is further coupled with grain growth predictions to evaluate the evolution of the solidification microstructure under different process conditions.

### 2.3. Nucleation and Grain Growth Modeling in Solidification

In twin-roll strip casting, solidification proceeds through two successive phases: the formation of nuclei and the subsequent growth of grains. Each phase is controlled by unique physical principles and requires different mathematical formulations. In the present study, appropriate models are employed to capture both nucleation and growth behavior. The nucleation rate is modeled as a function of undercooling and assumes heterogeneous nucleation behavior. Grain growth is described using empirical relations that couple local temperature gradients, growth velocity, and interface stability. These models are implemented in a fully coupled framework to capture the dynamic evolution of columnar and equiaxed grains under different casting conditions.

#### 2.3.1. Heterogeneous Nucleation Model

Heterogeneous nucleation, modeled using a Gaussian distribution, is incorporated within a continuous nucleation framework. The change in grain density during the nucleation process is described by a Gaussian distribution function with respect to undercooling. The model is expressed as:

(10) $$\frac{d_{n}}{d(\Delta T)}=\frac{n_{\max}}{\sqrt{2\pi }\Delta T_{\sigma }}\exp[-\frac{1}{2}{\left(\frac{\Delta T-\Delta T_{m}}{\Delta T_{\sigma }}\right)}^{2}]$$

where *d_n_* is the change in grain density for nucleation, expressed as surface density (m^−2^) or volumetric density (m^−3^); *d*(Δ*T*) is the increment of undercooling (K); Δ*T_m_* is the mean nucleation undercooling (K); Δ*T_σ_* is the standard deviation of undercooling (K); and *n*_max_ is the maximum nucleation density, expressed as surface density (m^−2^) or volumetric density (m^−3^).

#### 2.3.2. Growth Kinetics Model

Throughout solidification, the undercooling that develops at the solid–liquid interface can be represented as the sum of four different components:

(11) $$\Delta T=\Delta T_{c}+\Delta T_{t}+\Delta T_{r}+\Delta T_{k}$$

where Δ*T_c_* is the solutal undercooling, Δ*T_t_* is the thermal undercooling due to heat diffusion, Δ*T_r_* is the curvature undercooling at the solid–liquid interface, and Δ*T_k_* is the kinetic undercooling. Since solutal undercooling Δ*T_c_* plays a dominant role, the total undercooling can be approximated as Δ*T* ≈ Δ*T_c_*.

Based on the KGT model, Rappaz and Kurz [23] proposed a simplified relationship between dendrite tip growth velocity and undercooling, expressed as:

(12) $$\nu =\alpha {\left(\Delta T\right)}^{2}+\beta {\left(\Delta T\right)}^{3}$$

where *V* is the dendrite tip growth velocity, and *α* and *β* are the dendritic growth kinetic coefficients [24], which can be calculated using the material database in ProCAST. The calculation requires input parameters including the mass fraction of alloying elements, equilibrium partition coefficient *k*, slope of the liquidus line *m*, diffusion coefficient in the liquid phase *D*, and the Gibbs–Thomson coefficient *Γ*. These parameters are listed in Table 1.

In the simulation of the solidification microstructure, nucleation parameters include both surface nucleation and volumetric nucleation parameters. The modeling framework distinguishes between surface and volumetric nucleation: The former is assigned at the contact between molten steel and the cooling rolls, whereas the latter is distributed over the entire computational region. These parameters were determined based on data reported in the literature and are summarized in Table 2.

### 2.4. Computational Parameters and Boundary Conditions

#### 2.4.1. Computational Parameters

Low-carbon steel was chosen in this study to simulate the transport of mass and heat within the molten pool of twin-roll strip casting, and its main thermophysical properties are given in Table 3.

In describing the molten pool, three distinct phases are recognized: liquid, solid, and mushy. The mushy zone is defined as the intermediate region in which the solid fraction ranges from 0 to 1, and the solid fraction *f_s_* is determined according to the following formulation:

(13) $$f_{s}=\frac{T_{L}-T}{T_{L}-T_{S}}(T_{S}\leq T\leq T_{L})$$

**Table 3:** Physical properties of low-carbon steel.

**Property**	**Value**
Density (kg m^−3^)	7000
Thermal conductivity (W m^−1^ K^−1^)	36
Specific heat capacity (kJ kg^−1^ K^−1^)	680
Viscosity (Pa s)	0.0062
Latent heat of solidification (kJ kg^−1^)	270
Solidus temperature (K)	1768
Liquidus temperature (K)	1797

Where *T_L_* and *T_S_* are the liquidus and solidus temperatures, respectively. The density and thermal conductivity of the molten steel are affected by the phase state. To improve the accuracy of the numerical simulation, the density and thermal conductivity are defined using user-defined functions (UDFs) in ProCAST as follows:

(14) $$\rho \left\{\begin{array}{l} \rho_{L},T\geq T_{L}\\ \rho_{L}+f_{s}(\rho_{S}-\rho_{L}),T_{S}<T<T_{L}\\ \rho_{S},T<T_{S} \end{array}\right.$$

where *ρ_L_* is the density of liquid steel (7000 kg m^−3^), and *ρ_S_* is the density of solid steel (7200 kg m^−3^).

The effective thermal conductivity of low-carbon steel is defined as:

(15) $$k_{eff}\left\{\begin{array}{l} k_{L},T\geq T_{L}\\ k_{L}+f_{s}(k_{S}-k_{L}),T_{S}<T<T_{L}\\ k_{S},T\leq T_{S} \end{array}\right.$$

where *k_L_* is the thermal conductivity of liquid steel, set as 36 W/(m K), and *k_S_* is the thermal conductivity of solid steel, taken as 28.4 W/(m K).

#### 2.4.2. Boundary Conditions

The boundary conditions employed in this simulation are defined as follows:

(1) Inlet: The velocity at all inlets is assumed equal, calculated from the casting speed on the basis of mass balance. The inlet temperature, representing the molten steel entering the delivery system, is fixed at 1847 K.
(2) Free surface: The normal gradient of velocity at the free surface is set to zero, and the velocity component perpendicular to the surface is also set to zero. The heat transfer coefficient at the free surface is set as 20 W m^−2^ K^−1^.
(3) Roll surfaces: A no-slip condition is enforced at the cooling roll boundaries, with the velocity of the nodes assigned according to the casting speed. The thermal exchange between the molten steel and the rolls is modeled through a third-type (convective) boundary specification. The resulting heat flux can be written as:
  (16) $$-\lambda \frac{\partial T}{\partial n}=h_{roll}(T-T_{roll})$$
  where *λ* is the thermal conductivity of molten steel (W/(m·K)), *T* is the local temperature of the molten steel (K), *h_roll_* is the convective heat transfer coefficient between the molten pool and the roll surface (W m^−2^ K^−1^), and *T_roll_* is the surface temperature of the cooling roll (K).
(4) Side dam surfaces: The heat flux on the side dam surfaces is set as a constant value of 30,000 W m^−2^.
(5) Other wall surfaces: All remaining surfaces, excluding those explicitly defined above and the symmetry planes, are defined as solid wall boundary conditions and treated as adiabatic boundary conditions.

## 3. Results and Discussion

### 3.1. Effect of Side Hole Structure on Solidification Microstructure

**Figure 3:**
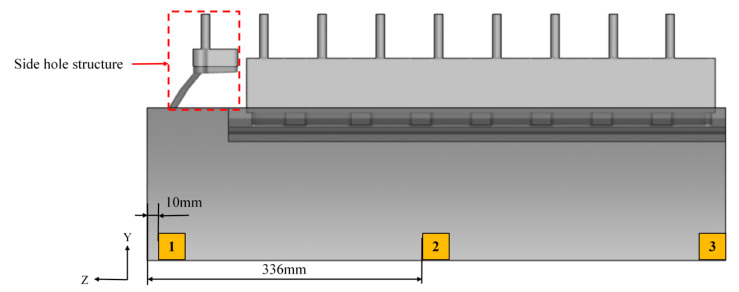
Sampling location for microstructure simulation in twin-roll strip casting.

**Figure 4:**
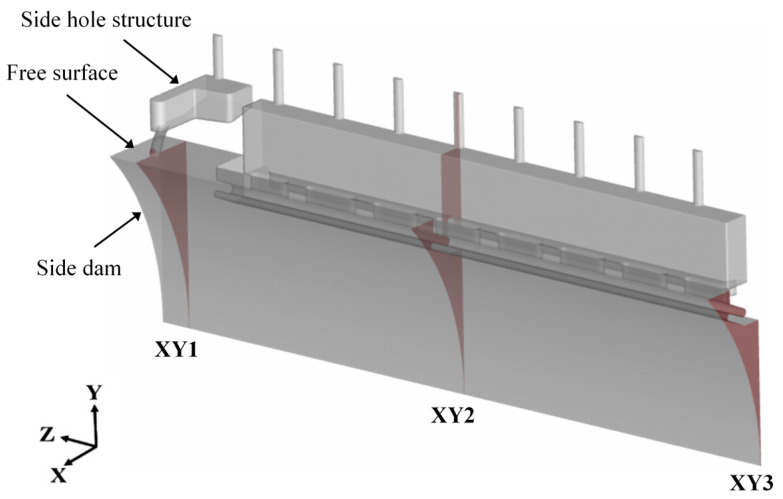
Quarter-section schematic of the molten pool showing sampling planes XY1, XY2, and XY3 in twin-roll strip casting.

To investigate the influence of the delivery system configuration on the solidification microstructure, quantitative analysis was conducted at representative locations across the strip width. The aim was to obtain the variation in grain morphology and size induced by the side hole structure. Figure 3 shows the sampling locations used for analyzing the as-cast microstructure predicted in twin-roll strip casting. Sampling position 1 is located 10 mm away from the side dam, and sampling position 2 is situated at one-quarter of the strip width direction (Z-axis), approximately 336 mm from the side dam. Sampling position 3 is positioned at the mid-width of the strip (1/2 along the Z-axis). Each sampling volume is 0.75 mm × 0.75 mm × 0.75 mm, enabling detailed observation of the microstructure distribution through the strip thickness. Figure 4 presents a schematic of the quarter-section of the molten pool, indicating the spatial relationship between the sampling sections (XY1, XY2, and XY3) and their corresponding positions across the strip width.

**Figure 5:**
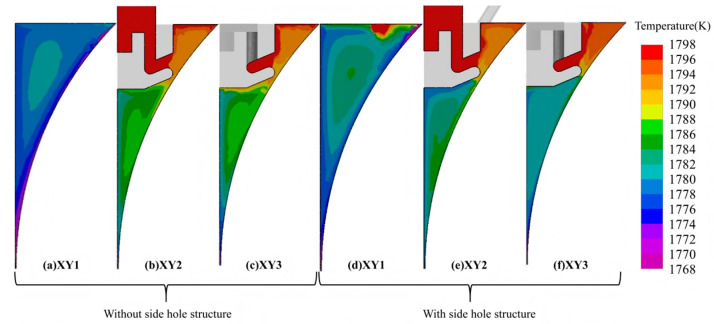
Temperature distribution across the section with different side hole structures.

**Figure 6:**
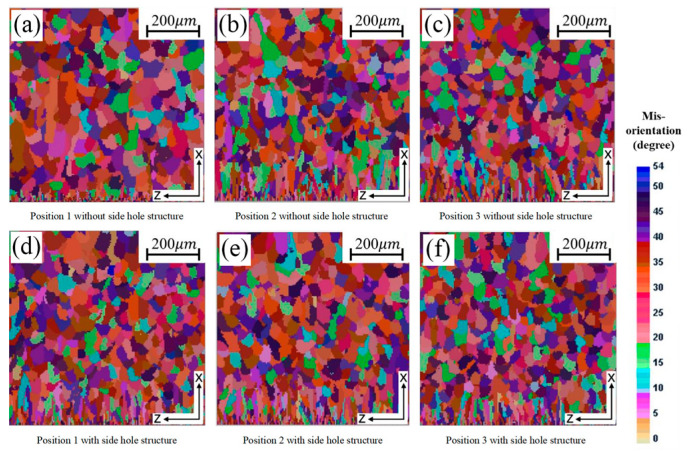
Simulation results of the microstructure at different positions with or without side hole structures.

Within the twin-roll strip casting process, variations in delivery system design exert a strong impact on fluid flow and heat transfer in the molten pool, which in turn govern the solidification process and the final microstructural features. Figure 5 presents temperature distributions for different sections under different side hole structures. In the absence of side holes in the delivery system, molten steel adjacent to the side dam is primarily supplied through lateral flow originating from neighboring zones of the pool. The flow in this area is largely governed by the backflow induced by the rotation of the cooling rolls. In addition, heat is dissipated through the side dam to the surroundings, resulting in a lower temperature and higher undercooling near the side dam. Consequently, heterogeneous nucleation occurs on the cooling roll surface in this region. Due to the high degree of undercooling, the nucleation rate is high, and grains compete to grow in multiple directions. As shown in Figure 6a, the solidified structure in this region is dominated by equiaxed grains, with indistinct columnar grains and coarse grain size. The average grain size in this zone is approximately 43.7 μm. In contrast, in other regions of the molten pool, the solidified structure consists of a fine-grained surface layer adjacent to the cooling roll, a sub-surface zone where columnar and equiaxed grains are interwoven, and a central equiaxed grain zone with a wide distribution of grain sizes. The average grain size in these regions is about 37 μm.

Table 4 summarizes the average grain size and grain density of the simulated solidification microstructures at different sampling positions. In conjunction with Figure 6, it can be observed that the microstructures at positions 2 and 3 (i.e., regions farther from the side dam) exhibit similar grain size, grain densities, and morphological characteristics. The average grain size at sampling positions 2 and 3 is 37.2 ± 0.1 μm, with a grain density variation of less than 1.5%. The observed similarity mainly arises from the flow distribution generated by the delivery system toward the cooling rolls, leading to a relatively uniform supply of molten steel across most of the pool, except near the side dams. As a result, the temperature distribution along the strip width becomes more uniform, leading to consistent microstructural features such as grain morphology and size.

Comparison of simulated microstructures at sampling position 1 reveals that introducing side holes into the delivery system facilitates the development of a distinct columnar grain region in the strip adjacent to the side dam. As shown in Table 4, when using the delivery system with the side holes, the grain size at position 1, corresponding to the strip region near the side dam, is reduced, resulting in a reduction in the average grain size in this region from 43.7 μm to 38.2 μm compared to the delivery system without side holes, while the grain density is significantly increased. This phenomenon arises because molten steel supplied via the side holes increases the temperature in the vicinity of the side dams. The elevated thermal field diminishes the degree of undercooling at the solidification front, which promotes preferential grain growth along the dominant temperature gradient and ultimately establishes a columnar grain zone. As the melt continues to cool and the undercooling increases to meet the nucleation conditions again, an equiaxed grain zone is subsequently formed.

### 3.2. Effect of Heat Transfer Coefficient on Solidification Microstructure

The cooling intensity of the rolls significantly affects the temperature distribution of molten steel in the pool, thereby influencing both the thermal gradient and the undercooling at the solidification front. As demonstrated in the previous section, the grain size and morphology of the solidification microstructures at sampling positions 2 and 3 show no substantial differences. Therefore, in this section and the following ones, only the solidification microstructure at sampling position 2 is selected for comparative analysis.

**Figure 7:**
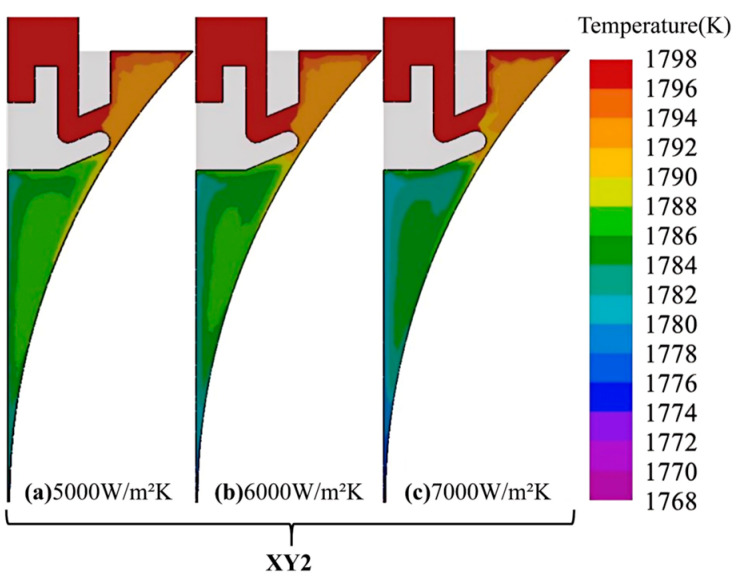
Temperature field at position 2 (XY2) under different *h_roll_*.

Figure 7 depicts the temperature field at position 2 (XY2 section) for different interfacial heat transfer coefficients between the rolls and the molten pool. The results indicate that changes in the coefficient have little influence on the upper pool region, while the lower portion is strongly affected. As the coefficient increases, the pool temperature decreases, leading to a higher solid fraction and an enlarged mushy zone, with the solidification endpoint moving upward. This trend arises from the intensified heat extraction between the molten steel and the cooling rolls at higher coefficients. However, an excessively high solid fraction and an enlarged mushy zone hinder melt flow, increasing the risk of solute segregation and crack formation in the strip.

**Figure 8:**
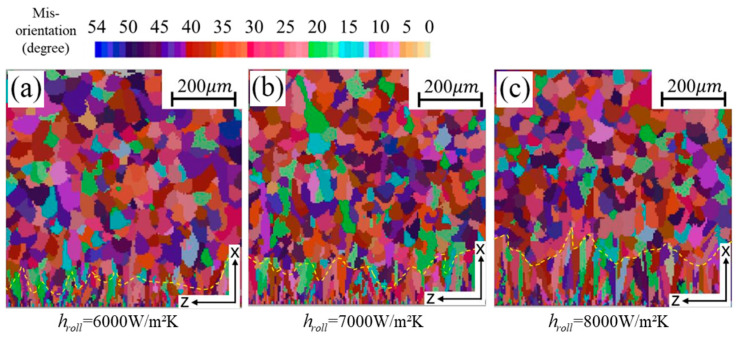
Simulated microstructure at position 2 under different *h_roll_*.

Figure 8 illustrates the simulated solidification structures at sampling position 2 under different values of the roll–pool heat transfer coefficient. As the coefficient rises, the columnar grain zone expands substantially, as marked by the yellow dashed boundaries. This phenomenon stems from the intensified thermal gradient that drives faster dendrite tip advancement at the solidification front. Nevertheless, the enhanced heat removal also reduces the overall pool temperature, which consequently increases the undercooling at the interface. This enhanced undercooling promotes nucleation and ultimately leads to the formation of an equiaxed grain zone at the strip center [27,28,29]. Table 5 summarizes the average grain size and grain density at sampling position 2 under different heat transfer coefficients between the molten pool and the cooling rolls. The average grain size is approximately 37 μm, and the grain density variation is less than 0.7%.The results indicate that variations in the heat transfer coefficient have a relatively minor influence on the average grain size and grain density.

### 3.3. Effect of Casting Speed on Solidification Microstructure

**Figure 9:**
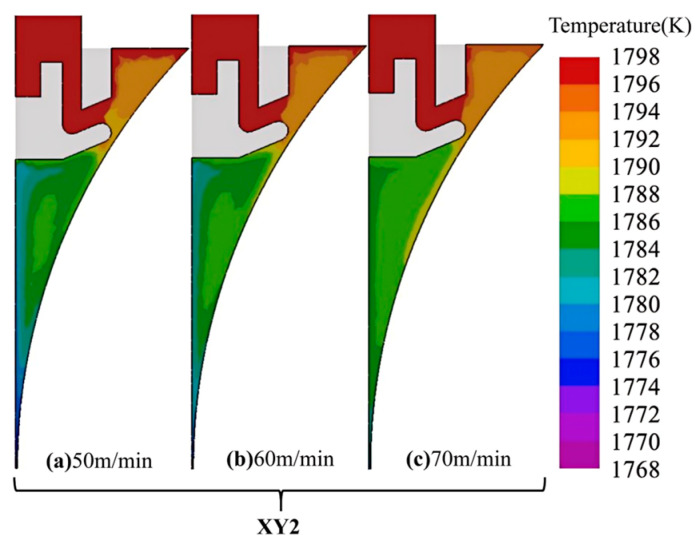
Temperature distributions at position 2 (XY2) under different casting speeds.

The flow of molten steel during twin-roll strip casting is largely governed by casting speed, which shapes the heat transfer environment and ultimately dictates the microstructural evolution and performance of the strip. Figure 9 illustrates the XY2-section temperature profiles under various casting speeds. When molten steel approaches the pool bottom, the contraction of the roll gap at the outlet prevents part of the liquid from discharging smoothly. This induces upward circulation and vortex structures that recirculate cooled steel from the bottom to the upper pool. As casting speed rises, the effective contact time between the melt and the cooling rolls diminishes, thereby reducing heat removal.

Meanwhile, the presence of vortices promotes mixing between the cooler bottom steel and the hotter upper steel, enhancing temperature uniformity within the pool. This uniformity is beneficial for reducing the risk of segregation and crack formation in the final strip. Additionally, higher casting speeds intensify molten steel flow, further improving thermal uniformity. However, excessive casting speeds may shift the solidification end point downward, potentially compromising strip quality and increasing the risk of breakout.

**Figure 10:**
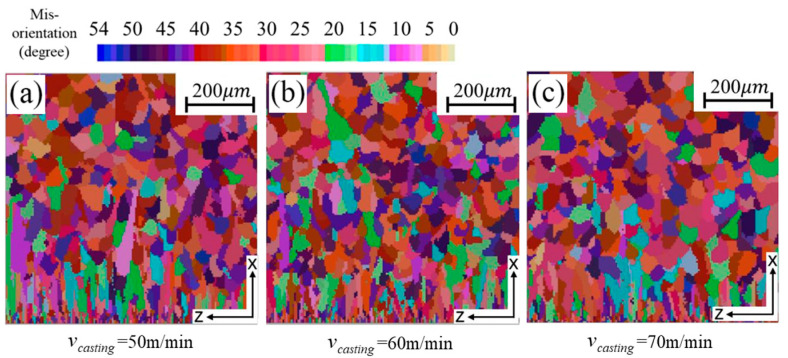
Simulated solidification microstructure at position 2 under different casting speeds.

Figure 10 illustrates the simulated microstructures at sampling position 2 under varying casting speeds. The results show that as casting speed increases, the columnar grain zone becomes smaller. This effect is mainly attributed to the higher melt flow velocity, which intensifies impingement on the solidified shell near the cooling rolls. The stronger flow enhances nucleation, encouraging equiaxed grain formation while suppressing columnar growth. In addition, greater casting speeds shorten the contact time between the melt and the rolls, reducing heat extraction and raising the pool temperature. The elevated temperature may induce partial remelting of dendrites, further restricting columnar grain development.

Table 6 presents the average grain size and grain density at sampling position 2 under different casting speeds. Increasing the casting speed from 50 m min^−1^ to 70 m min^−1^ reduces the area of the columnar grain zone, while the average grain size decreases slightly (by less than 0.5 μm), and the grain density increases accordingly. However, the overall magnitude of these changes remains limited.

### 3.4. Comparison Between Experimental and Simulated Solidification Microstructures in Twin-Roll Strip Casting

**Figure 11:**
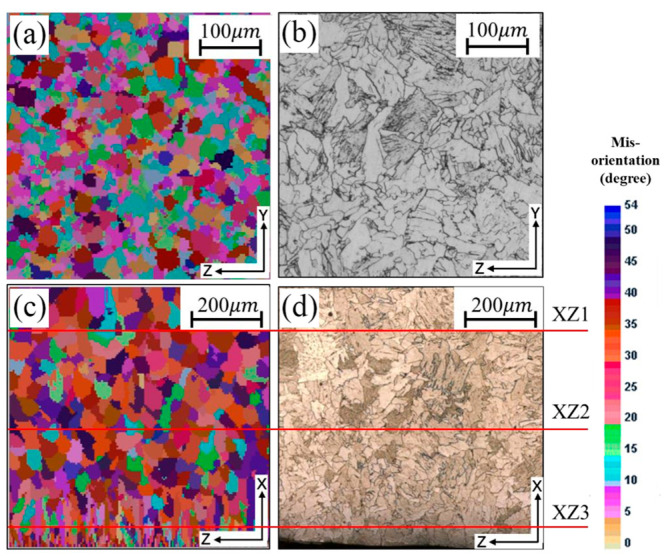
Microstructure of twin-roll strip casting: (**a**,**c**) simulation results, (**b**,**d**) experiment results.

The thin strip samples used in this work were taken from areas far from the side dams. As shown in Figure 6 and Table 4, these samples exhibited uniform morphology and grain size. Thus, the simulation results at position 2 were selected for comparison with metallographic experiments. Figure 11 compares simulated and observed microstructures. Grain size distributions were measured using ImageJ software, where three horizontal and three vertical lines were applied to each image and average diameters calculated by the line intercept method. The average grain diameter in the simulated solidification microstructures was obtained using the post-processing module of ProCAST. To investigate the grain size distribution along the strip thickness (XZ section), straight lines were also extracted from near-edge, sub-edge, and central regions in Figure 11c,d. Grain sizes at each position were calculated using the same method, with red lines indicating the intercept locations. The comparison of average grain sizes from simulation and experiment is shown in Table 7.

Figure 11a,b show the simulated and experimental solidification microstructures along the strip width direction (YZ section) of the twin-roll cast strip. The microstructure is composed of a mixed arrangement of equiaxed and columnar grains, with significant variation in grain size across the section. Figure 11c,d present the simulated and experimental results along the strip thickness direction (XZ section). It is evident that the grain size near the strip edges, close to the cooling rolls, is relatively small, while toward the center, the grain size increases, primarily forming coarse columnar and equiaxed grains in an interwoven structure. Table 7 compares the average grain sizes obtained from the simulation and the metallographic experiment. It can be seen that the simulated grain sizes are close to those measured experimentally. The distribution trend of grain size along the XZ section also agrees with Figure 11c,d, where the average grain size is smallest near the edge and increases progressively toward the strip center [30]. Overall, Figure 11 and Table 7 demonstrate that the simulated solidification microstructures agree closely with the experimental observations in both grain size and morphology, thereby validating the reliability of the simulations.

## 4. Conclusions

In this study, the solidification behavior and microstructure evolution during twin-roll strip casting were investigated through a combination of physical experiments and numerical simulations. The following main conclusions were obtained:

(1) The solidification microstructure in twin-roll strip casting consists of three distinct regions: an equiaxed grain zone near the cooling roll surface, a columnar grain zone growing from the equiaxed base, and a coarse equiaxed grain zone in the center. The relative proportions of the equiaxed and columnar zones are affected by process parameters.
(2) For the model adopted in this study, the solidification microstructures in regions far from the side dam exhibit consistent grain size, density, and morphology. The average grain size at sampling positions 2 and 3 is 37.2 ± 0.1 μm, with a grain density variation of less than 1.5%. The delivery system with side holes promotes the formation of a distinct columnar grain zone near the side dam area, resulting in a reduction in the average grain size in this region from 43.7 μm to 38.2 μm compared to the delivery system without side holes.
(3) As the heat transfer coefficient between the molten pool and the cooling rolls increases from 5000 W m^−2^ K^−1^ to 7000 W m^−2^ K^−1^, the area of the columnar grain zone expands significantly. However, this change has little effect on the average grain size and grain density, with the average grain size remaining close to 37 μm and the grain density variation being less than 0.7%. In contrast, increasing the casting speed from 50 m min^−1^ to 70 m min^−1^ reduces the area of the columnar grain zone, while the average grain size decreases slightly (by less than 0.5 μm), and the grain density increases accordingly.

## Figures and Tables

**Table 1:** Calculation parameters of dendrite tip growth kinetics coefficients [23].

Element	Mass Fraction	k	m/(K %^−1^)	D/(m^2^ s^−1^)	*Γ*/(K m)
C	0.03	0.19	−78.0	20	3.0 × 10^−7^
Mn	0.60	0.76	−4.9	2.4	
Si	0.16	0.77	−7.6	2.4	
S	0.03	0.05	−38.0	4.5	
P	0.00	0.23	−34.4	4.7	

**Table 2:** Nucleation parameters and kinetic growth coefficients of the microstructure [2,25,26].

Parameter	α/(m sK^−2^)	β/(m sK^−3^)	ΔT_s,max_/(K)	ΔT_s,σ_/(K)	n_s,max_/(m^−2^)	ΔT_v,max_/(K)	ΔT_v,σ_/(K)	n_v,max_/(m^−3^)
Value	2.7 × 10^−6^	3.8 × 10^−4^	0.5	0.1	2.5 × 10^−10^	2.5	1	4.7 × 10^10^

**Table 4:** Average grain size and grain density of the simulated microstructure.

Grain Structure Statistics	Side Hole Structure	Sampling Position		
		1	2	3
Average grain size (μm)	No	43.7	37.1	37.3
Average grain size (μm)	Yes	38.2	37.3	37.1
Grain density (mm^−2^)	No	1213.7	1702.2	1719.0
Grain density (mm^−2^)	Yes	1614.7	1695.2	1699.8

**Table 5:** Average grain size and grain density of the simulated microstructure under different *h_roll_*.

Heat Transfer Coefficient (W m^−2^ K^−1^)	6000	7000	8000
Average grain size (μm)	36.9	37.1	37.2
Grain density (mm^−2^)	1697.1	1702.2	1691.6

**Table 6:** Average grain size and grain density of the simulated microstructure under different casting speeds.

Casting Speed (m min^−1^)	50	60	70
Average grain size (μm)	37.6	37.1	36.8
Grain density (mm^−2^)	1688.7	1702.2	1722.5

**Table 7:** Simulated and experimental mean grain size.

Location	Simulated Grain Size (μm)	Experimental Grain Size (μm)
Width direction (YZ section)	19.8	20.3
Thickness direction (XZ section)	37.0	38.2
XZ1	50.5	52.7
XZ2	44.5	44.1
XZ3	20.4	26.5

## Data Availability

The original contributions presented in this study are included in the article. Further inquiries can be directed to the corresponding author.
